# Unanticipated diagnosis of skeletal muscle Kaposi sarcoma: a case report

**DOI:** 10.11604/pamj.2020.37.158.26412

**Published:** 2020-10-14

**Authors:** Harshini Udayakumar, Venkatraman Indiran, Prabhakaran Madurai Muthu

**Affiliations:** 1 Department of Radiodiagnosis, Sreebalaji Medical College and Hospital, CLC Works, Road, Chromepet, Chennai, Tamilnadu 600044, India

**Keywords:** Kaposi sarcoma, skeletal muscle sarcoma, atypical Kaposi sarcoma

## Abstract

Kaposi sarcoma (KS) is a cancer, characteristically manifesting as red or purple patches of abnormal tissue growing subcutaneously around the mouth, nose, and throat. Primary musculoskeletal KS is a never reported as skeletal muscles sarcomas are first differentials. Pertaining to the musculoskeletal system complicity of KS, African and classic KS lesions are inclined to manifest lesion in the peripheral skeleton. On the other hand AIDS-related KS routinely involves the maxillofacial bones and/or axial skeleton. KS distinguishably involves the tempo-parietal bones, paranasal sinus, hands and feet and other facial bones. Asymmetric involvement of the bones by KS is the rule. Though reported, involvement of the joints in KS is unusual. Skeletal muscle involvement has only sparingly been reported in AIDS-related KS patient. A primary KS of the skeletal muscle in an otherwise normal patient with no skin manifestations has never been reported thus far. The occurrence of KS in any atypical site may pose as difficulty to diagnose it. It is important for the radiologist to acquaintance with the spectrum of imaging manifestations of KS in various affected organs. Particularly in asymptomatic patients, lesions go unrecognized on routine imaging studies (e.g. KS on plain x-ray films) and clinicians are unwary of their existence. Awareness that KS can occur in any of these unusual locations may help avoid potential misdiagnosis with serious consequences (e.g. spinal cord compression) and/or mis-management.

## Introduction

Primary musculoskeletal Kaposi sarcoma in a non-immunocompromised or a non-acquired immune deficiency syndrome (AIDS) patient has not been yet reported in literature.

## Patient and observation

**Figure 1:**
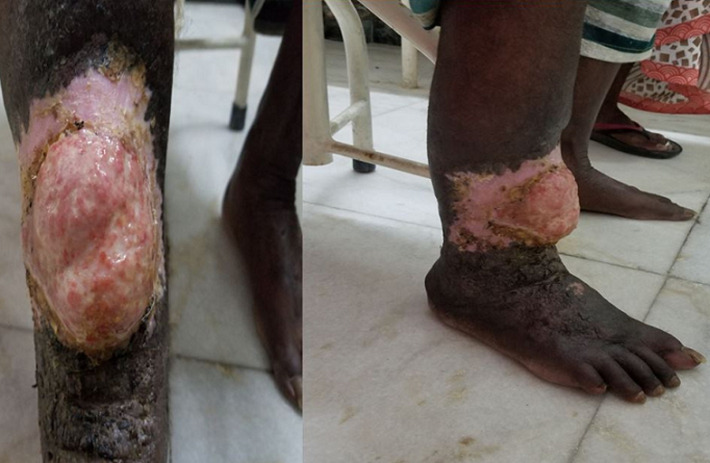
clinical photography of the right leg shows a well demarcated erythematous firm lesion with surrounding nodular areas peripherally and accompanying lymphedema of the entire leg and foot

**Figure 2:**
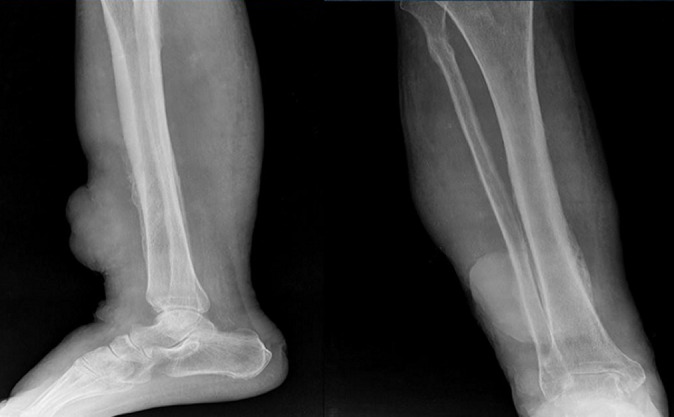
X-ray anteroposterior and lateral projection of the right leg shows a lobulated soft tissue swelling in the anterior aspect of the lower one third of the leg with few calcific areas within and associated periosteal reaction the adjacent bones of the leg

**Figure 3:**
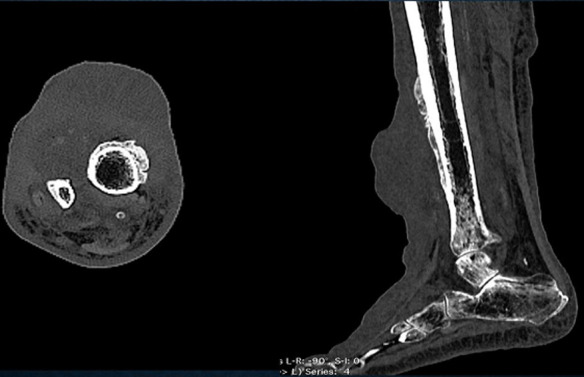
computerised tomography (CT) axial and sagittal reconstruction images of the same patient shows areas of soft tissue edema, heterogeneous linear densities and few areas of faint calcification in the region of the lobulated skin lesion; thick unicortical periosteal reaction of the tibia for a, short segment; adjacent to the lesion is noted

A 70 year old man of Indian origin presented to the outpatient department with complaints of pain, limited mobility and swelling over anterior aspects of the right leg, above the ankle for three months. He gave a history of recurrent ulcer over the same region. There was no history of loss of sensation, no history of trauma, no comorbid illness, no history of fever, no gastrointestinal/respiratory symptoms. Clinical examination of the right leg showed a well demarcated erythematous firm lesion with nodular surrounding areas and accompanying lymphedema of the distal foot. Thorough clinical examination of the entire body revealed no other lesion. There was no past history of similar lesions anywhere in the body. No other skin/oral lesions were identified. Additional investigations showed he was not immunocompromised or did not suffer from any sexually transmitted diseases. Plain radiograph of the leg (anteroposterior and lateral views) showed a lobulated soft tissue swelling in the anterior aspect of the lower one third of the leg with few calcific areas within and associated periosteal reaction the adjacent bones of the leg. Computerised tomography (CT) of the same patient shows areas of soft tissue edema, heterogeneous linear densities and few areas of faint calcification in the region of the lobulated skin lesion. Thick unicortical periosteal reaction of the tibia for a short segment; adjacent to the lesion was noted. Magnetic resonance imaging (MRI) showed findings similar to the CT findings and shows no bone marrow involvement. The lesion appeared iso to hypo intense on T1 weighted images and hyper intense on T2 weighted images and was located within the skeletal muscle. Surrounding perilesional edema was noted. There was no bone marrow involvement (Figure 1, Figure 2, Figure 3). A biopsy of the lesion was performed and showed histopathological features of Kaposi sarcoma.

## Discussion

**Figure 4:**
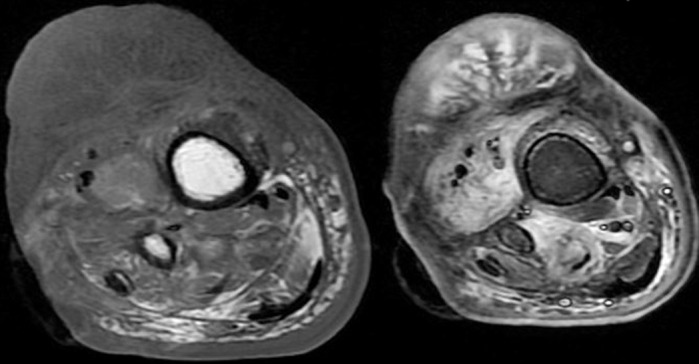
MRI of the same patient (axial T1 and T2 STIR images) is consistent with the CT findings and shows no bone marrow involvement

**Figure 5:**
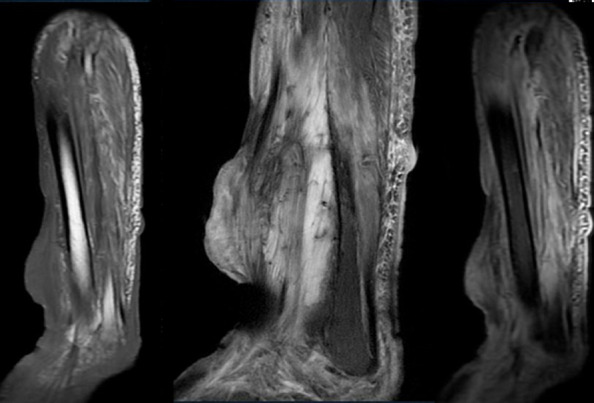
MRI of the same patient (sagittal T1 and T2 and STIR images) shows a T1 iso to hypo intense lesion within the skeletal muscle; T2 hyperintense edema is noted; no bone marrow involvement is seen

**Figure 6:**
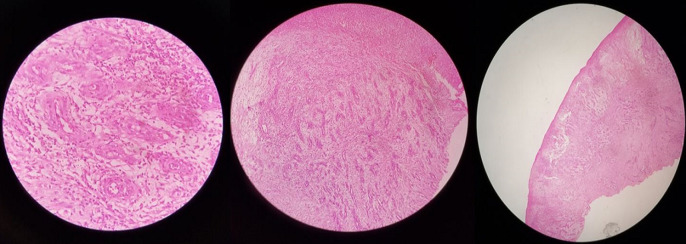
histopathological examination of the biopsy specimen shows stratified squamous epithelium below the dermis, consistent with tumor tissues and shows proliferation of spindle cells around the blood vessels with individual spindle cells showing nuclear atypia and mild hyper chromatosis; they also form slit like, spaces and show a few inflammatory cells; features suggestive of low grade sarcoma - Kaposi sarcoma

Musculoskeletal involvement in KS is uncommon and if at all, occurs secondarily due to local extension from the skin surface lesion. All epidemiological forms of KS have been shown to involve the musculoskeletal system. They include AIDS-related KS, classic KS, African (endemic) KS, and infrequently transplanted-associated KS [1]. The largest study on KS involving the musculoskeletal system included about 66 cases that were published earlier [2]. Of these cases, three of them from the review included KS manifestations within the skeletal muscle. All of the patients with skeletal muscle manifestations were AIDS-related KS in that study. Osseous lesions in KS are more commonly encountered than primary skeletal muscle lesions alone. Involvement of the bone marrow, without causing any osseous lesion, is far more common in AIDS-related KS. Conventional radiography can shows bone involvement with erosion to frank destruction of bone as well as periosteal reaction. On CT shows soft-tissue like masses are seen. MR imaging depicts bone marrow abnormalities and soft-tissue masses. KS lesions develop as due to a number of co factors: human herpes virus 8 (HHV8), alteration of immunity (immunocompromised state), and angiogenic or inflammatory-factors. This helps explain how KS is also seen to develop from traumatized tissue or from pemphigus vulgaris lesions [3]. African form of KS and classic form of KS lesions commonly seem to involve the peripheral skeleton, whereas AIDS-related KS on the contrary commonly involves the axial skeleton. These include the ribs, sternum, pelvis and vertebrae in decreasing order of frequency. Maxillofacial bones are also uncommonly involved [4,5]. The paranasal sinuses, the maxilla, mandible, hard palate and temporal bone are some of the skull bones of involvement in KS. Few other bones that are reported to be involved by KS include the vertebrae most commonly T11 to L4, the ribs, the pelvis and the sternum, along with the long bones such as the humerus, femur, radius, ulna, tibia and the fibula. Small bones of the hands namely the metacarpals are involved. Talus, calcaneum and 3, 4, 5 metatarsals are the common bones of the feet that are involved in KS. Asymmetric involvement of the bone by KS is the rule observed so far. Joint involvement is unusual, but has been reported [6] (Figure 4, Figure 5, Figure 6).

## Conclusion

Clinically the differentials pertaining to dermatological examination were pyogenic granuloma, baciliary angiomatosis, acroangio dermatitis, hemangiomas and other melanocytic proliferations. The radiological differential diagnosis depending on imaging and history included a broad spectrum and were soft tissue sarcomas, osteomyelitis, bacillary angiomatosis which may also exhibit skin lesions, bone erosions, and soft-tissue masses, mycobacterial infections, pyogenic granuloma and lymphoma. The biopsy of the lesion was performed and was reported as features suggestive of low grade sarcoma-Kaposi sarcoma. The occurrence of KS in any of the above mentioned atypical sites may prove difficult to arrive at KS diagnosis, particularly if the patients are relatively asymptomatic, lesions can go unrecognized on routine imaging studies (e.g. on plain X-ray films osseous KS can be missed), and/or clinician's awareness of their existence [7]. Being mindful of the idea that KS can occur in any of the mentioned unusual locations may avoid the potential misdiagnosis which could have serious consequences (e.g. compression of the spinal cord) and/or any mismanagement that could occur (e.g. life-threatening haemorrhage can occur when a biopsy of laryngeal KS is done) [7]. KS involvement of infrequent sites must be considered in the differential diagnoses as KS may mimic common lesions.
